# Associations between cerebral and systemic endothelial function in migraine patients: a post-hoc study

**DOI:** 10.1186/1471-2377-11-146

**Published:** 2011-11-15

**Authors:** Denis Perko, Janja Pretnar-Oblak, Mišo Šabovič, Marjan Zaletel, Bojana Žvan

**Affiliations:** 1 University Medical Centre Ljubljana, Department of Neurology, Zaloška cesta 2, 1000 Ljubljana, Slovenia; 2 University Medical Centre Ljubljana, Department of Internal Medicine, Zaloška cesta 7, 1000 Ljubljana, Slovenia

## Abstract

**Background:**

There is a growing interest in the role of the endothelium in migraine. Recently, our group showed differences in endothelial function between the anterior and posterior cerebral circulation in healthy subjects, reduced vasodilatatory capacity of the posterior cerebral circulation and unimpaired systemic endothelial function in migraine patients without comorbidities. However, the relationship between cerebral and systemic endothelial function and the anterior and posterior cerebral endothelial function in migraine patients is still not clear.

**Methods:**

We compared cerebral and systemic endothelial function through post-hoc linear regression analysis of cerebrovascular reactivity (CVR) to L-arginine between the middle cerebral artery (MCA) and flow-mediated vasodilatation (FMD) of the right brachial artery and the posterior cerebral artery (PCA) and FMD in migraine patients without comorbidities and in healthy subjects. The anterior and posterior cerebral endothelial function was also compared using post-hoc linear regression analysis between CVR to L-arginine in the MCA and the PCA.

**Results:**

No significant correlation was found between CVR to L-arginine in the MCA and FMD and in the PCA and FMD in migraine patients with aura (p = 0.880 vs. p = 0.682), without aura (p = 0.153 vs. p = 0.179) and in healthy subjects (p = 0.869 vs. p = 0.662). On the other hand, we found a significant correlation between CVR to L-arginine in the MCA and PCA in migraine patients with aura (p = 0.004), without aura (p = 0.001) and in healthy subjects (p = 0.002). Detailed analysis of the linear regression between all migraine patients and healthy subjects did not show any difference in the regression coefficient (slope) (p = 0.382). However, a significant difference in curve elevation (intercept) was found (p = 0.002).

**Conclusions:**

Our study suggests that the endothelial function in the cerebral and systemic circulation might be different in migraine patients without comorbidities, while that of the anterior and posterior cerebral circulation might be coupled. These results could improve understanding of endothelial function in migraine patients without comorbidities.

## Background

In recent years it has been proposed that migraine patients have endothelial dysfunction [1]. However, both our group and that of Vanmolkot have found unimpaired systemic endothelial function in migraine patients without comorbidities [2,3]. Instead, we have shown that endothelial dysfunction in migraine patients without comorbidities could be limited to the posterior cerebral circulation [4]. Vanmolkot et al. also referred to the lack of studies comparing cerebral and systemic endothelial function in the same patients [3]. Therefore, it is still unclear whether there is an association between cerebral and systemic endothelial function in migraine patients.

The only study comparing cerebral and systemic endothelial function simultaneously is that of Pretnar-Oblak et al., who concluded that cerebral and systemic endothelial function might not be closely associated in patients with lacunar infarctions [5]. However, this study compared endothelial function in the anterior cerebral and systemic circulation. No study comparing the posterior cerebral and systemic endothelial function has yet been undertaken.

One of the findings of our previous study was related to higher endothelial function in the posterior cerebral circulation compared to that in the anterior circulation in healthy subjects [6]. Despite this finding, and the fact that migraine patients might have endothelial dysfunction in the posterior cerebral circulation, it is still unclear whether the endothelial function in the posterior and middle cerebral artery territories is associated in migraine patients.

In our previous studies, the methods of cerebrovascular reactivity (CVR) to L-arginine and flow- mediated vasodilatation (FMD) were used to assess the anterior and posterior cerebral and systemic endothelial function [7-14]. We also measured carotid intima-media thickness (IMT) obtained the medical history and performed physical and neurological examinations, and routine laboratory tests. With inclusion criteria of normal values of IMT and exclusion of vascular risk factors, only migraine patients without comorbidities and healthy subjects without known underlying vascular disease or risk factors were included in these studies.

The aims of our study were as follows: I) to compare endothelial function in the anterior and posterior cerebral circulation with the systemic endothelial function in migraine patients without comorbidities; II) to compare the endothelial function in the anterior and posterior cerebral circulation in migraine patients without comorbidities.

Although these comparisons have not yet been performed, this knowledge could be important for understanding the endothelial role of different vascular beds in migraine patients without comorbidities.

## Methods

This post-hoc study was performed using data obtained from previous studies with the primary aim of comparing endothelial function in the anterior and posterior cerebral with the systemic endothelial function and the secondary aim of comparing endothelial function in the anterior and posterior cerebral circulation in migraine patients without comorbidities. Forty migraine patients and twenty healthy subjects participated in two previous studies, which were approved by the National Medical Ethics Committee of the Republic of Slovenia. All subjects gave written informed consent before being included in the study. Migraine patients were diagnosed according to the International Headache Society criteria (2^nd ^edition) [15]. Healthy subjects were randomly recruited from hospital staff and acquaintances after completion of a questionnaire. Migraine patients were randomly recruited from a headache clinic. All subjects had a normal somatic and neurological examination. Migraine patients were divided into two groups, 20 patients with migraine with aura (MwA) and 20 patients without aura (MwoA). The median monthly headache attack frequency in migraine patients was 2.0 ± 1.1. The three groups were matched for gender (4 males and 16 females in each group) and age (healthy 33.4 ± 7.5 years, MwA 34.1 ± 8.3 years, MwoA 34.3 ± 7.7 years). None of the subjects in the control group was suffering from headache when the study was conducted and none had migraine or other headache. Migraine patients had the last migraine episode more than 24 hours before the investigations were conducted.

The major exclusion criteria were: history of cardiovascular disease, arterial hypertension (systolic blood pressure (SBP) > 140 mm Hg or diastolic blood pressure (DBP) > 90 mmHg), body mass index (BMI) < 18 and ≥ 25 kg/m^2^, hypercholesterolaemia (total cholesterol > 5.5 mmol/L), diabetes, IMT > 1.0 mm, pregnancy or lactation and regular use of vasoactive drugs (except triptane or other transient vasoactive antimigraine drugs).

Colour-coded duplex sonography of the carotid and vertebral arteries was performed in all patients. IMT was measured according to the Mannheim Intima-Media Thickness Consensus on both sides 2 cm below the bifurcation on the far wall of the common carotid artery [16]. The distance between the characteristic echoes from the lumen-intima and media-adventitia interfaces was measured. The final IMT value was based on the mean value of three maximal IMT measurements. Subjects with plaques (focal structures that encroached into the arterial lumen of at least 0.5 mm or 50% of the surrounding IMT value or demonstrated a thickness > 1.5 mm) were excluded from the study.

FMD of the right brachial artery was performed according to the recommendations of Corretti et al. in a quiet room under constant conditions between 7.30 and 10.30 am after a fasting period of at least 10 hours [17]. A high-resolution ultrasound system with a 10-MHz linear array transducer located 2 to 10 cm above the antecubital fossa was used. The brachial artery was scanned in the longitudinal section, and the end-diastolic mean arterial diameter was measured at the end of the diastole period, incident with the *R*-wave on the simultaneously recorded electrocardiogram. A hyperaemic flow increase was then induced by inflation of a blood pressure cuff to a pressure of 50 mm Hg higher than the measured systolic blood pressure for 4 minutes. The hyperaemic diameter was recorded within 1 minute after cuff deflation and the final scan was performed 4 minutes later. FMD was expressed as the percentage change in the artery diameter after reactive hyperaemia relative to the baseline scan.

CVR to L-arginine was simultaneously measured in the anterior and posterior cerebral circulation. For this purpose the middle (MCA) and the posterior cerebral artery (PCA) were chosen. The experiment consisted of a 10-minute baseline period, a 30-minute intravenous infusion of 100 mL 30% L-arginine, and a 10-minute period after L-arginine application. The mean arterial velocity (v_m_) in the MCA was recorded through the left temporal acoustic window at a depth of 50 to 60 mm, and in the PCA through the right temporal acoustic window at a depth of 50 to 60 mm, with a mechanical probe holder maintaining a constant probe position. TCD Multi-Dop X4 software was used to determine v_m _during the 5-minute baseline period and the 5-minute period after L-arginine infusion. CVR to L-arginine in the PCA and the MCA was expressed as the percentage change in the v_m _after stimulation with L-arginine.

The variables FMD and CVR were statistically analysed by the statistic software SPSS 18.0 and GraphPad. For this purpose linear regression analysis was used to analyse possible correlations between the variables of FMD and CVR in each of the groups: all migraine patients, with aura, without aura, and healthy subjects. In addition, linear regression analysis was used to test the significance of the regression coefficients and curve elevations between migraine patients (all, with aura, without aura) and healthy subjects.

## Results

All subjects had normal values of SBP, DBP, BMI, total cholesterol, HDL, LDL, triglycerides, IMT, and glucose (Table 1) [4].

**Table 1:** Biochemical and morphological parameters of healthy subjects, MwA and MwoA

	Healthy	MwA	MwoA
**Total cholesterol (mmol/L)**	4.79 ± 0.74	4.68 ± 0.68	4.41 ± 0.87
**HDL cholesterol (mmol/L)**	1.65 ± 0.36	1.64 ± 0.30	1.61 ± 0.33
**LDL cholesterol (mmol/L)**	2.79 ± 0.52	2.67 ± 0.72	2.35 ± 0.76
**Triglycerides (mmol/L)**	0.79 ± 0.34	0.89 ± 0.35	0.82 ± 0.23
**Serum glucose (mmol/L)**	4.97 ± 0.50	4.66 ± 0.35	4.73 ± 0.49
**SBP (mmHg)**	115.3 ± 7.9	116.2 ± 10.5	118.9 ± 11.8
**DBP (mmHg)**	77.0 ± 6.6	77.6 ± 5.6	79.2 ± 6.9
**BMI (kg/m^2^)**	20.8 ± 1.5	21.3 ± 2.3	22.2 ± 2.6
**IMT (mm)**	0.39 ± 0.07	0.39 ± 0.07	0.40 ± 0.06

MwA = migraine with aura, MwoA = migraine without aura, SBP = systolic blood pressure, DBP = diastolic blood pressure, BMI = body mass index, IMT = intima-media thickness

**Figure 1:**
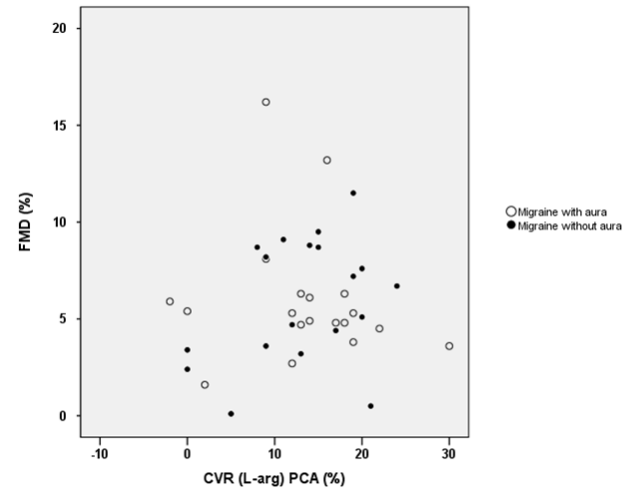
**Scatter plot of comparison between CVR to L-arginine in the PCA and FMD**. Scatter plot of comparison between cerebrovascular reactivity (CVR) to L-arginine in the posterior cerebral artery (PCA) and brachial artery flow-mediated dilation (FMD) in migraine patients with and without aura (p = 0.582; r^2 ^= 0.008).

**Figure 2:**
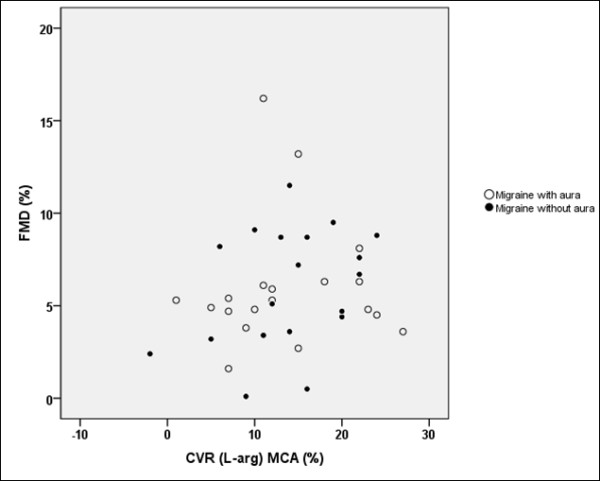
**Scatter plot of comparison between CVR to L-arginine in the MCA and FMD**. Scatter plot of comparison between cerebrovascular reactivity (CVR) to L-arginine in the middle cerebral artery (MCA) and brachial artery flow-mediated dilation (FMD) in migraine patients with and without aura (p = 0.287; r^2 ^= 0.031).

First, we analysed the correlation between FMD and CVR to L-arginine. We did not find any significant correlation between FMD and CVR to L-arginine in the MCA (p = 0.287; r^2 ^= 0.031; Figure 1) and between FMD and CVR to L-arginine in the PCA (p = 0.582; r^2 ^= 0.008; Figure 2) in all migraine patients. Similarly, we did not find any significant correlation between FMD and CVR to L-arginine in the MCA in MwA (p = 0.880; r^2 ^= 0.001) and MwoA (p = 0.153; r^2 ^= 0.117), or between FMD and CVR to L-arginine in the PCA in MwA (p = 0.682; r^2 ^= 0.010) and MwoA (p = 0.179; r^2 ^= 0.104), nor did we find any significant correlation between FMD and CVR to L-arginine in the MCA (p = 0.869; r^2 ^= 0.002) and FMD and CVR to L-arginine in the PCA (p = 0.662; r^2 ^= 0.011) in healthy subjects.

**Figure 3:**
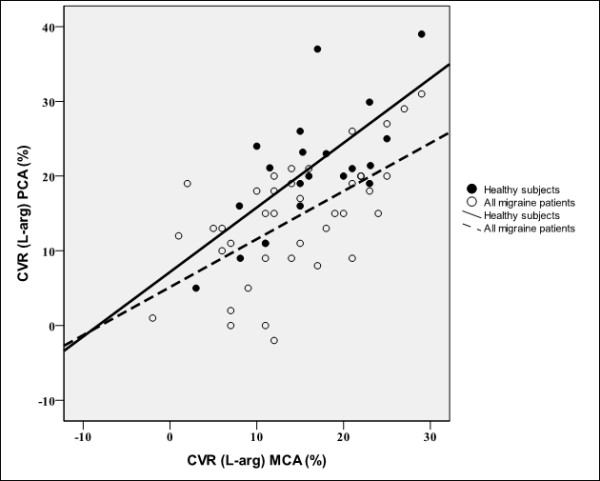
**Correlation between CVR to L-arginine in the MCA and the PCA**. Correlation between cerebrovascular reactivity (CVR) to L-arginine in the middle cerebral artery (MCA) and the posterior cerebral artery (PCA) in all migraine patients (p < 0.001; r^2 ^= 0.377) and healthy subjects (p = 0.001; r^2 ^= 0.481).

In the next step we analysed the correlation between CVR to L-arginine in the PCA and the MCA. We found a significant positive correlation between CVR to L-arginine in the PCA and the MCA in all migraine patients (p < 0.001; r^2 ^= 0.377; Figure 3), MwA (p = 0.008; r^2 ^= 0.333), MwoA (p = 0.001; r^2 ^= 0.441) and in healthy subjects (p = 0.001; r^2 ^= 0.481), but we did not find any significant difference in regression coefficients (*b*) between all migraine patients (b = 0.642) and healthy subjects (b = 0.866) (p = 0.382), MwA (b = 0.584) and healthy subjects (p = 0.342) and MwoA (b = 0.725) and healthy subjects (p = 0.624). However, we found a significantly higher rate of curve elevation (intercept) in the group of healthy subjects compared to that in the group of all migraine patients (p = 0.002), MwA (p = 0.01) and MwoA (p = 0.007).

## Discussion

The main and original finding of our post-hoc study is that we did not find any correlation between CVR to L-arginine in the anterior and posterior cerebral circulation and FMD in migraine patients without comorbidities and healthy subjects. In other words, our results showed that endothelial function in the cerebral circulation probably differs from that in the systemic circulation under physiological conditions as well as in neurovascular disorders such as migraine.

There are almost no studies comparing cerebral and systemic endothelial function. To the best of our knowledge, the only study to perform such a comparison is that of Pretnar-Oblak et al., who reported an absence of any association between CVR to L-arginine in MCA and FMD in patients with lacunar infarctions [5]. However, they did not compare CVR to L-arginine in the PCA and FMD, and their patients obviously had vascular risk factors, which altered endothelial function in the cerebral and systemic circulation. It is important to highlight the supposition that migraine is a systemic vascular disorder. Several studies have shown that migraine is associated not only with cerebrovascular events, but also with disorders of the coronary, retinal and peripheral vasculature [1]. However, in many studies the authors did not exclude vascular risk factors, or perhaps they excluded many vascular risk factors but did not evaluate IMT, a morphological marker of the early atherosclerotic process and associated endothelial dysfunction, which consequently might have biased their findings [18-23]. Nevertheless, the results of our study and that of Pretnar-Oblak et al. are in accordance with the supposition that endothelial function is not uniform throughout the arterial system. This difference could be present between organs and different vascular beds within the same organ, even in the absence of diseases known to affect endothelial function [24-26]. Furthermore, the belief that atherosclerosis is the main pathophysiological process in coronary disease - and to lesser extent in cerebrovascular diseases - might also reflect this fact.

It must be emphasized that cerebral endothelial function in healthy subjects or in patients with endothelial dysfunction cannot be predicted on the basis of evaluation of the systemic endothelial function. Only by simultaneous comparison of both endothelial functions can we evaluate the association between the cerebral and systemic circulation. Since studies have shown differences in endothelial function between organs, one might expect these differences also to exist between the cerebral and systemic circulation in healthy subjects. However, this may not apply to patients with endothelial dysfunction of the cerebral and systemic circulation. For this reason we evaluated endothelial function only in migraine patients without comorbidities.

Linear regression analysis of CVR to L-arginine between the MCA and the PCA showed positive correlations in healthy subjects and migraine patients with and without aura, without any significant differences in regression coefficients (slopes) between healthy controls and migraine patients (Figure 2). These results could suggest a coupling of the endothelial function in the anterior and posterior cerebral circulation in both healthy subjects and migraine patients. Indeed, the whole brain must be supplied with blood, regardless of which parts needs a higher blood flow. The anterior and posterior cerebral circulation is anatomically coupled by the circle of Willis. However, the physiological factors involved in the coupling are largely unknown. The results of animal studies suggest that the endothelium in the anterior and posterior circulation responds differently to the precursor of nitric oxide (NO) and inhibitors of NO synthetase (NOS) and that the posterior cerebral circulation is subject to more basal vasomotor control to NO than the anterior cerebral circulation [27-29]. In addition, our previous study showed the possibility that the endothelium of the posterior cerebral circulation has a higher vasodilatatory capacity compared to the anterior cerebral circulation [6].

Nevertheless, the regulation of regional blood flow in the territory of the posterior cerebral artery is probably altered in migraine patients [4]. This assumption has again been shown by a significant difference in curve elevation (intercept) between migraine patients and healthy controls (Figure 3). Therefore, it could be speculated that migraine patients have reduced vasodilatatory capacity in the posterior cerebral circulation. Despite this finding, our present study implies endothelial coupling between the anterior and posterior cerebral circulation, not only in healthy subjects but also in migraine patients.

Our study has some limitations. The study population was relatively small; hence our conclusions must be treated with some caution. The methods, FMD and CVR to L-arginine, have some limitations and they probably reflect different aspects of induced vasoreactivity, hence they cannot be simply compared. The hypercapnic response before and after L-arginine infusion could further improve understanding of cerebral endothelial function [30]. However, the mechanisms of CO_2_-produced vasodilatation are not entirely known, and CO_2_-induced vasodilatation is only partially related to NO-dependent mechanisms [31]. Since the aims of this and our previous studies were to evaluate and compare endothelium-dependent vasodilatation of the cerebral and systemic circulation in migraine patients, we did not evaluate endothelium-independent vasodilatation of the brachial artery. We believe that this would not give additional information about the role of the endothelium in vasoreactivity. Another limitation is that, with the exclusion of migraine patients with thickened IMT, we did not take into account the possibility that migraine itself might induce thickening of IMT and consequently impair vasoreactivity. However, we believe that this explanation is less likely. It must also be emphasized that evaluating endothelial function involves not only evaluating vasoreactivity but also the inflammatory and coagulatory milieu [32,33]. However, the tests of both milieus are nonspecific.

The results of this study are different from those obtained in our previous studies since neither the evaluation of the relationship between FMD and CVR to L-arginine in the PCA and the MCA nor the evaluation of the relationship between CVR to L-arginine in the PCA and the MCA have been performed in our previous studies or in any other study in which migraine patients were included [2,4,6].

Considering all these findings, it would be interesting to discover whether evaluation of the cerebral endothelial function in migraine patients could be useful in identifying migraine patients at higher risk of experiencing ischaemic cerebral events.

## Conclusion

Taking into account the presented analysis and our previous results, the following can be concluded in migraine patients without comorbidities: I) the cerebral and systemic circulations probably have different endothelial functions; II) endothelial function in the anterior and posterior cerebral circulation might be coupled.

## Competing interests

The authors declare that they have no competing interests.

## Authors' contributions

Each author made significant and substantial written contributions to this manuscript. All authors read and approved the final version of the manuscript.

## Pre-publication history

The pre-publication history for this paper can be accessed here:

http://www.biomedcentral.com/1471-2377/11/146/prepub
